# Single Molecule Bioelectronics and Their Application to Amplification-Free Measurement of DNA Lengths

**DOI:** 10.3390/bios6030029

**Published:** 2016-06-24

**Authors:** O. Tolga Gül, Kaitlin M. Pugliese, Yongki Choi, Patrick C. Sims, Deng Pan, Arith J. Rajapakse, Gregory A. Weiss, Philip G. Collins

**Affiliations:** 1 Department of Physics and Astronomy, University of California at Irvine, Irvine, CA 92697, USA; 2 Department of Physics, Polatlı Faculty of Science and Arts, Gazi University, Polatlı 06900, Turkey; 3 Department of Chemistry, University of California at Irvine, Irvine, CA 92697, USA; 4 Department of Physics, North Dakota State University, Fargo, ND 58108, USA; 5 Department of Molecular Biology and Biochemistry, University of California at Irvine, Irvine, CA 92697, USA

**Keywords:** DNA polymerase, carbon nanotube sensors, single molecule enzymology, DNA sequencing

## Abstract

As biosensing devices shrink smaller and smaller, they approach a scale in which single molecule electronic sensing becomes possible. Here, we review the operation of single-enzyme transistors made using single-walled carbon nanotubes. These novel hybrid devices transduce the motions and catalytic activity of a single protein into an electronic signal for real-time monitoring of the protein’s activity. Analysis of these electronic signals reveals new insights into enzyme function and proves the electronic technique to be complementary to other single-molecule methods based on fluorescence. As one example of the nanocircuit technique, we have studied the Klenow Fragment (KF) of DNA polymerase I as it catalytically processes single-stranded DNA templates. The fidelity of DNA polymerases makes them a key component in many DNA sequencing techniques, and here we demonstrate that KF nanocircuits readily resolve DNA polymerization with single-base sensitivity. Consequently, template lengths can be directly counted from electronic recordings of KF’s base-by-base activity. After measuring as few as 20 copies, the template length can be determined with <1 base pair resolution, and different template lengths can be identified and enumerated in solutions containing template mixtures.

## 1. Introduction

In populations of organisms, cells, or molecules, atypical individuals can exert disproportionate roles. In the least consequential cases, these individuals merely nudge the average activity or phenotype of a population; but in more severe cases, they turn on entirely new responses or enable pathways with pathological consequences [1]. For example, a single cancerous cell can initiate a tumor formation or provoke an immune response [2,3,4]. At an even finer scale, a single errant or mutant biomolecule can disrupt a cell signaling pathway or inappropriately activate transcription [5,6]. Understanding cause and effect in a population is very difficult when single individuals can change the behavior of the overall system, and addressing this challenge requires a detailed study of protein function at the individual level.

Over the past two decades, tremendous progress has been made developing techniques that can address the challenge of characterizing the activity of single molecules. Resolving individual molecules and tracking their dynamic activities has grown into a major subfield at the forefront of biophysical research, leading to a better understanding of the functions of molecular motors, signaling proteins, and pharmaceutical inhibitors [7,8,9,10,11]. Analysis of many individual molecules also provides opportunities to understand heterogeneity within a population and the transition from normal function into malfunction. For example, observation of enzyme catalysis at the single-molecule level has observed that otherwise-identical molecules can have very different turnover rates. By resolving individual chemical events and comparing the reaction trajectories of enzymes, single-molecule techniques reveal the variable dynamics of efficiency, processivity, and kinetics that are all hidden in ensemble averages. Furthermore, details about intramolecular distances and forces [12,13,14], molecular orientation [15,16], substrate binding and release mechanisms [17,18,19], and substrate preferences [20,21,22] can each be revealed.

These underlying motivations have driven the development of a variety of single-molecule experimental techniques. Single-molecule fluorescence has led the way as a versatile method for tracking energy transfer and the relative motions of submolecular domains [23,24], and single-molecule fluorescence is the basis for at least one commercial DNA sequencing technique [25]. In addition, force-based techniques have used magnetic tweezers, optical tweezers, or scanning probes to perturb molecules and measure mechanical responses [26,27]. Although optical and mechanical techniques have been well represented, electronic transduction has generally been missing from the single-molecule toolbox, with the notable exception of patch-clamp techniques for monitoring individual ion channels [28]. However, the recent development of electronic devices with length scales smaller than 10 nm promises to bring electronic measurement modes to this field. Future researchers may be able to choose among optical, mechanical, and electronic transduction methods and select techniques or combinations of complementary techniques, which are best suited to a particular research question.

Achieving this vision of a versatile single-molecule toolkit requires that single-molecule electronic devices become well-controlled and accessible. To be practical for biomolecule research, these devices must operate near room temperature in physiologically relevant, conductive fluids, and have nanometer-scale conduction channels that are well-matched to the size of individual molecules. Nature’s elegant solution to these requirements involves transmembrane proteins that fold to define interior, nm-scale pores through which ionic currents can flow [29]. The general idea of ionic currents in nanopores has been successfully extended to synthetic, inorganic barriers like SiN or polymer films [30,31]; a DNA sequencing technology based on arrays of nanopores has been commercialized by Oxford Nanopore Technologies [32,33]. Aside from ionic currents, the scanning tunneling microscope provides one example of a conduction channel for electrons that has single-molecule sensitivity. Although arrays of tunneling microscopes have proven impractical, precision deposition of thin insulating films has helped extend the principle of tunneling to arrays of stable, solid state devices that can compete with nanopores for sensitivity [34,35]. Another competing new technology is based on nanoscale transistors made from Si nanowires [36,37,38,39,40] or carbon nanotubes [41,42,43,44,45,46], conducting wires that are sensitive enough to transduce the motions of single molecules and their dynamics.

**Figure 1:**
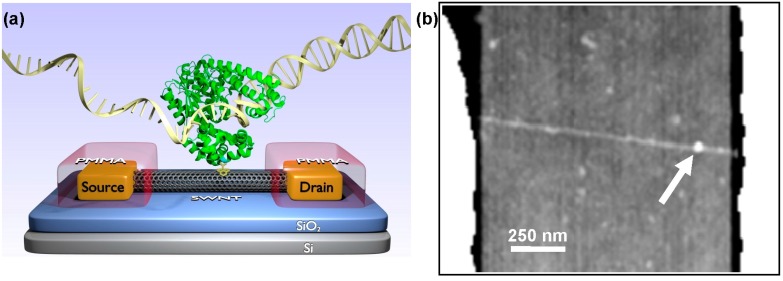
(**a**) Schematic of a single-walled carbon nanotube field effect transistor (SWNT-FET) functionalized with a single copy of a protein. This device depicts the Klenow Fragment of DNA polymerase I converting a single-stranded DNA template into a double-stranded helix. A pyrene-maleimide (**yellow**) is shown linking the protein to the SWNT. (**b**) Atomic force microscopy image of a typical SWNT-FET device after functionalization confirms attachment of a single protein (**arrow**).

For this special issue on carbon nanotube sensing, we review the fabrication and operation of single-molecule carbon nanotube-based biosensing devices. Abundant research over the past decade has demonstrated the biosensing capabilities of carbon nanotubes, and recently this sensitivity has been extended to single-molecule electronics. Specifically, experiments have attached single enzymes to single-walled carbon nanotube field-effect transistors (SWNT-FETs, Figure 1a) and demonstrated the versatile ability of tracking an enzyme’s conformational states and revealing distributive and processed enzyme movements. The electronic nature of the FET technique has immediate benefits such as microsecond time resolution and long-duration capabilities, which are both advantages over fluorescence for studying the conformational dynamics and processivity of a single molecule. SWNT-FETs functionalized with T4 lysozyme [21,46,47], cAMP-dependent protein kinase A [48], and the Klenow Fragment of DNA Polymerase I (KF) [49,50] have all been successfully used to study each enzyme’s dynamic variability and demonstrate the capabilities of the SWNT-FET approach.

The remainder of the paper is organized into three sections. Section 2 describes methods of fabricating SWNT-FETs and labeling them with single active molecules like enzymes. Section 3.1 summarizes example results from SWNT-FETs sensing the activity of DNA polymerases processing single-stranded DNA templates. As an illustrative example of the general SWNT-FET technique, the DNA polymerase devices are particularly useful for two reasons. First, the range of possible DNA templates and both native and chemically modified starting materials for DNA polymerization make this a rich and complex experimental system. Second, accurate sensing of DNA polymerase activity has practical applications in DNA sequencing; nearly all single-molecule techniques have been applied to this challenge, and published data is thus available for direct comparison with the SWNT-FET results. Section 3.2 describes new analysis of the accuracy of the SWNT-FET technique and the challenges that must be overcome for these devices to be successfully deployed in DNA sequencing.

## 2. Materials and Methods

### 2.1. Fabrication and Functionalization of SWNT-FETs

The initial demonstration of field-effect transistors based on SWNTs [51,52] and silicon nanowires [36] laid the foundation for the later development of hybrid devices incorporating biomolecules. Early research revealed the general sensitivity of these one-dimensional (1-D) FETs [41,42], which could then be coated with sensitizing agents to produce a variety of chemoresistive responses for biosensing [36,53]. However, continuous coatings fail to take advantage of the unique properties that make 1-D FETs unmatched by other types of chemical sensors. Namely, 1-D channels allow scattering at a single location to be communicated to distant electrodes. The intrinsic amplification of a scattering site in an otherwise pristine, 1-D channel allows single molecular events to produce output signals that can be monitored by relatively simple control electronics.

Taking advantage of this unique sensitivity requires a strategic functionalization approach. To avoid ensemble averaging of the signals from multiple independent molecules, sensitization should be limited to one active site. The success of this strategy was first demonstrated by introducing single point defects into SWNT-FETs, which imbued each device with a chemical degree of freedom while also localizing sensitivity to the modified position [54]. Researchers have monitored the kinetics of individual molecular reactions [44] and DNA binding events [45,55] using defect sites; however, these defects enhance sensitivity without substantial benefits in signal-to-noise and at the cost of damaging the carbon lattice. Consequently, non-covalent surface functionalization remains preferable if it can be controlled with single-site precision. As described below, non-covalent schemes have been successfully diluted to the point that SWNT-FETs have either zero or one sensitizing molecule, according to Poisson statistics [21,46]. These techniques are the focus of this review.

Aside from functionalization, the fabrication of single-molecule devices follows protocols for SWNT-FETs that have become standard in the field. SWNTs can be deposited onto surfaces by spin coating or spraying from solutions, by dry transfer from other substrates, or by direct synthesis in place. The latter technique exposes SWNTs to the least chemical processing, manipulation, and damage; so it is advantageous for achieving precise control of single active sites. We spin coat a water-soluble solution of Fe_30_Mo_84_ catalyst nanoparticles [56] onto 4′′ silicon wafers, diluted to achieve the desired SWNT density. The wafers are then processed in a chemical vapor deposition (CVD) furnace [57]. The particles are oxidized to remove their stabilizing ligands (air, 5 min at 700 °C), reduced to a catalytically active form (10% H_2_ in Ar, 5 min at 940 °C), and then exposed to a carbon source that initiates SWNT growth (15% CH_4_ and 10% H_2_ in Ar, 5 min at 940 °C). The catalyst density is set to achieve a uniform density of ~0.01 SWNTs/μm^2^, so that arrays of electrodes patterned on the wafer by photolithography will contact, on average, only one SWNT.

In our process, three-terminal FETs are achieved by using degenerately doped (p++) silicon wafers with a 250 nm thermal oxide. The SiO_2_ is a good promoter of catalyst activity and SWNT growth, and it serves as a gate dielectric between the SWNTs and the underlying wafer. Source and drain contacts are defined on top of the SWNTs using a bilayer undercut resist (S-1808 on LOR-1A, Microchem), followed by electron beam evaporation of a thin sticking layer (0.7 nm Ti) and electrode metal (30 nm Pt). After liftoff, the exposed FETs are coated with passivating layers of Al_2_O_3_ and/or polymer resist (poly(methyl methacrylate), PMMA in Figure 1a) to protect the SWNTs and SWNT-metal interfaces from the environment. After fabrication, the entire wafer is mapped to determine the resistance, transconductance, and type of each electrical connection. For the purposes of successful prototyping, extra care is taken to investigate devices individually. Stable, noise-free devices with resistances <5 MΩ are selected for investigation by scanning electron microscopy and atomic force microscopy (AFM) to ensure that each is comprised of an individual SWNT free from particles or other obvious contaminants.

Biofunctionalization of the SWNT-FETs is a three-step process. First, a small window is opened in the protective passivation layer over the SWNT-FET channel. Using either optical or electron-beam lithography, a small portion (~1 μm) midway between the source and drain electrodes is removed to expose the SWNT sidewall to the environment while keeping the rest of the device protected. Next, the devices are soaked in a solution of a non-covalent linker molecule like *N*-(1-pyrenyl)maleimide (saturated solution in EtOH, Sigma-Aldrich, St. Louis, MO, USA). The linker is chosen to have a pyrene or other aromatic polycyclic functional group to adhere to the SWNT sidewall via π-π stacking. After rinsing (phosphate-buffered saline, 0.1% Tween-20, Acros Organics, Geel, Belgium) to remove excess linkers, the device is soaked in a buffered aqueous solution containing the biomolecule to be attached to the SWNT. Numerous bioconjugation chemistries are possible, including attachments through unnatural amino acids [58], HaloTags [59], or even the terminal His tags used for protein purification [60]. We have selected a linker containing a maleimide group to take advantage of stable thioether bond formed with the thiol of a surface-exposed cysteine [61,62]. With the help of mutagenesis, proteins with a single exposed cysteine can be designed, expressed, and purified for attachment to the SWNT using pyrene-maleimides; often several single cysteine variants must be prepared to identify an appropriate site for bioconjugation to the SWNT.

The successful creation of single-molecule devices occurs by preparing multiple SWNT devices in parallel and tailoring the final bioconjugation step to yield only 0.2 to 0.3 molecules per device. Protein incubation is performed for 15 to 60 min using protein concentrations of 50 to 500 nM, where the optimum conditions for a particular protein are determined empirically using AFM to directly image the conjugation yield. An example image of one protein attachment is shown in Figure 1b. The optimum incubation parameters are specific to each protein due to the accessibility of the cysteine attachment site, the protein’s tendency to aggregate, and the concentration dependence of undesirable nonspecific adsorption. Alternate conjugation chemistries would involve additional considerations. After a final rinse to remove unbound protein, the devices are ready for measurement or storage. After successful measurements, devices are always imaged by AFM to verify the presence of one active protein.

The fabrication of SWNT-FETs is an active field that continues to be refined. In addition to the methods described here, investigators are successfully parallelizing large arrays of SWNT-FETs for digital electronics applications [63,64] and solving the interfacial materials problems that cause excess noise and resistance [65,66]. The non-covalent biofunctionalization scheme described here is easily modified to other linkage schemes and readily adapted to surfaces containing many devices; with automated microfluidic dispensers it can be expanded to arrays of devices having different proteins or protein variants. Thus, many opportunities exist for combining best practices and expanding proof-of-principle single-molecule devices into functional arrays for different commercial uses.

### 2.2. Operation of Single-Molecule SWNT-FETs

**Figure 2:**
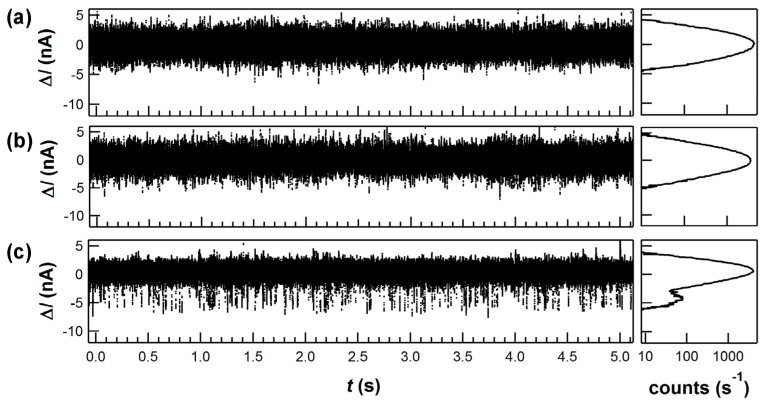
Example ∆*I*(*t*) signals and signal histograms generated by KF in a solution containing: (**a**) 1 nM poly(dA)_42_ homopolymer template; (**b**) template with 10 μM non-complementary dGTP; and (**c**) template with 10 μM complementary dTTP. Both (**a**) and (**b**) are indistinguishable from the baseline noise fluctuations observed from a SWNT, either with or without KF attachments. Only combinations of a successful KF attachment and a solution containing template and complementary dNTPs produced the type of additional excursions shown here.

After fabrication, chemical activity of the attached protein is transduced into fluctuations in the source-drain current *I*(*t*) flowing in the SWNT channel (Figure 2). *I*(*t*) measurements are usually performed with the active portion of the device submerged in a buffered aqueous solution containing the protein’s binding partners and other reaction co-factors. Typical *I*(*t*) values of 10 to 100 nA require a current preamplifier with an appropriate bandwidth. For example, a Keithley 428 amplifier has a 15 µs rise time at 10^7^ V/A gain, which is a suitable choice for resolving dynamics down to 100 µs.

Successful measurements also require stable control of the electrode and electrolyte potentials. An electrochemical bipotentiostat can provide complete control of the three-terminal FET and its electrolyte gate, but simpler methods are sufficient. We typically use DC sources to apply up to 100 mV to the drain electrode, 0 V to the Si wafer back gate, and a modest biasing voltage (−0.3 to +0.3 V) to the electrolyte using a Pt wire as a counter electrode. Except for the Pt wire, the electrical connections are wirebonds or probing needles located outside the electrolyte.

*I*(*t*) signals from the current amplifier are digitized and saved for real-time or offline analysis. Typical data sets of 10 min are usually sufficient to determine the average kinetics of a single molecule, though data may be collected for much longer durations. To test a molecule with different analyte concentrations or combinations as shown in Figure 2, it is important to rinse a device with buffer until molecular activity appears to cease. Depending on the degree of nonspecific surface adsorption, a device may need to sit in buffer for up to 10 min before rinsing returns the device to its baseline current *I*(*t*). Testing a molecule with many analytes, and performing proper control measurements for each case, usually involves dozens of rinses that benefit from integrating the data acquisition with an automated fluid delivery system (e.g., SmartSquirt, Automate Scientific). Presumably, nonspecific adsorption could be further reduced by surface blocking techniques used in other biosensing or surface analytical techniques, but these methods and their effects on single-molecule sensitivity have not yet been explored with SWNT-FET devices.

**Figure 3:**
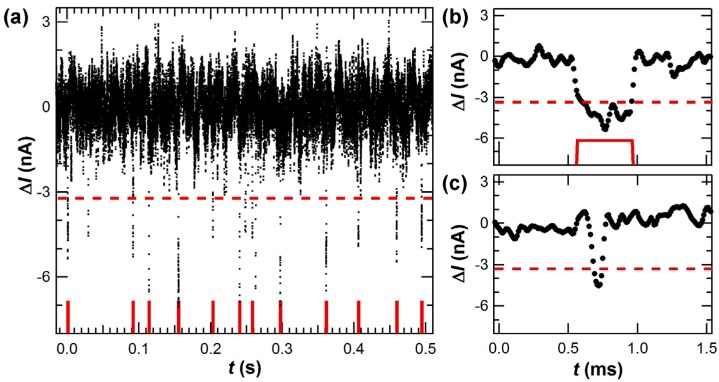
(**a**) ∆*I*(*t*) excursions and event enumeration (**red**) at higher magnification. Individual excursions were counted as dNTP incorporations if they exceeded a threshold determined by the baseline 1/f noise (dashed lines) and if their duration exceeded 100 μs (*i.e.*, 10 data points). (**b**,**c**) Magnified plots of the first two ∆*I*(*t*) excursions in (**a**) demonstrate examples of: (**b**) a base incorporation event; and (**c**) an excursion rejected for having a short, unresolved duration. A 40 μs amplifier rise time limited analysis to the longer events, but better time resolution might reliably identify brief ∆*I*(*t*) excursions caused by noncatalytic KF motions.

Finally, data analysis is an important part of the proper operation of these devices. One advantage of the SWNT-FET technique is its ability to continuously monitor the full reaction trajectory of a molecule over minutes, hours, or days. However, SWNT-FETs generate noise with a 1/f spectrum [67,68], meaning that long-duration measurements also exhibit large-magnitude baseline fluctuations. AC coupling or other highpass filtering successfully eliminates the lowest-frequency wanderings and emphasizes just the deviations Δ*I*(*t*) from the DC average. Figure 3a illustrates the typical noise levels after filtering, and it depicts the threshold level used to distinguish fluctuations that are attributable to KF with certainty. The filter cutoff must be chosen carefully to avoid biasing the analysis of protein activity. For example, substantial 1/f noise in the 0.1 to 10 Hz frequency band directly overlaps with the enzymatic activity of DNA polymerase, which has average turnover rates of 20 to 40 s^−1^ amid stochastic pauses lasting 1 s or more. The overlap of the desired signal with SWNT noise requires more sophisticated filtering techniques such as the open source analysis solutions vbFRET [69] and NoRSE [70] or, for the most challenging analyses, other nonlinear, machine learning techniques [71,72]. At present, reliable enumeration of enzyme information from long data records remains a challenging task that has only been partly automated.

To provide a specific example, Section 3 is focused on single-molecule measurements of the Klenow Fragment (KF) of DNA polymerase I, a moderately processive and well-characterized polymerase from *E. coli*. For these experiments, a single-cysteine variant of exonuclease-defective KF (D355A/E357A/L790C/C907S) [73] was engineered using site-directed mutagenesis followed by overexpression and purification from *Escherichia coli* (*E. coli*) [49,50]. KF was attached to SWNT-FETs as described above using 500 nM protein in a standard DNA polymerase I activity buffer (20 mM Tris, 50 mM NaCl, 10 mM MgCl_2_, 100 μM TCEP, pH 8.0). Separately, a fluorescence-based ensemble assay confirmed activity of this variant.

To measure KF activity, devices were submerged in an activity buffer containing 10 μM of dNTPs (Fisher Scientific) and 0.5 to 100 nM of DNA templates. The templates were synthesized by fusing an M13 priming site to different sequences of overhanging bases. The M13 priming site of each template was hybridized to an M13 forward primer in a 1:1 stoichiometric ratio by heating to 95 °C for 5 to 10 min followed by slow-cooling to room temperature. Following hybridization, overhanging bases for each template were composed of homopolymeric sequences as short as 10 bases and more varied sequences as long as 104 bases. Most of the measurements reported here are focused on the repeating patterns (CTTT)_9_, (CTTT)_10_, and (CTTT)_11_.

## 3. Results

### 3.1. Single-Molecule Activity of DNA Polymerase (KF)

The primary function of DNA polymerases like KF is the replication or repair of DNA. Under the direction of a single-stranded DNA template, the polymerase incorporates dNTPs into a complementary template strand. The nascent strand lengthens base-by-base as the polymerase accepts an incoming dNTP and catalyzes its addition to the new strand’s 3′-hydroxyl terminus [74]. A minimum scheme [75] for each cycle of this process requires multiple kinetic steps and checkpoints. In the first step, the enzyme’s so-called “thumb” domain binds a primer-template DNA to form a binary open complex E·DNA_n_. Successful recognition and binding of a complementary dNTP allows the enzyme’s “fingers” subdomain to snap closed on the activated ternary complex E*·DNA_n_·dNTP. This conformational transition has been directly observed by numerous smFRET-based experiments [76,77,78,79,80,81,82,83] and inferred from co-crystal X-ray structures [84,85]. As it completes incorporation of the new base, the polymerase translocates one position along the template to begin a new cycle or else dissociates from the template [86,87].

This complex cycle makes monitoring KF activity an excellent test of the SWNT-FET single-molecule technique. Many of KF’s critical steps have remained hidden, including dNTP recognition, error checking, and translocation, though the enzyme has been investigated using a range of techniques [88]. The precise timing of KF’s reopening, the variability of KF’s kinetic rates, and its dissociation probabilities each remains unknown or contested [89,90,91]. Part of this problem arises from the fact that the most common single-molecule method, Förster resonance energy transfer (smFRET), has limited time resolution for very fast events [92] and no capacity for long-duration monitoring of a single, FRET-labeled molecule [93]. The SWNT-FET technique, on the other hand, has been demonstrated with 2 µs resolution [72]; the approach can also be used to observe long, time-varying reaction trajectories [21,48] because the enzyme of interest is essentially permanently attached to the device. In a typical 10-min measurement, a single KF will incorporate thousands of dNTPs into many template molecules, whereas no smFRET measurement has measured more than three successive replications by the same KF molecule [77].

To be especially sensitive to dNTP incorporation events, KF was engineered to attach to SWNT-FETs on the back side of the active fingers subdomain (Figure 1a), using the single-cysteine bioconjugation protocol described above [85]. After attachment, KF activity was monitored electronically by placing the device in different solutions of DNA template and dNTPs. Figure 2 shows example *I*(*t*) records acquired under various experimental conditions. Stochastic excursions Δ*I*(*t*) occurred at 10 to 40 s^−1^ when template and complementary dNTPs were both present in the measurement solution, consistent with smFRET measurements of KF replication [78]. Δ*I*(*t*) excursions of this type disappeared when the complementary dNTPs were absent or replaced by non-complementary dNTPs [49], or when template was removed from the solution. These types of control experiments helped to confirm that Δ*I*(*t*) excursions like these were unique fluctuations associated with the incorporation of new bases and not simply the random motions of KF in solution.

**Figure 4:**
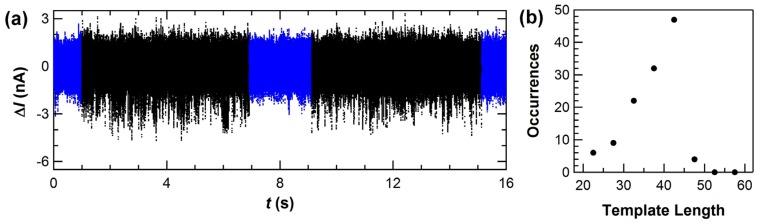
(**a**) Template concentrations below 5 nM introduced additional pauses (**blue**) in the records of KF activity. The pauses represent diffusional waiting times for template arrival and association. Each uninterrupted sequence of ∆*I*(*t*) excursions (**black**) represents the processing of one template strand. (**b**) Distribution of apparent lengths observed using a low concentration of the homopolymer (dC)_42_ template, as determined by counting the individual ∆*I*(*t*) excursions in a 10-min data set containing 135 uninterrupted sequences.

In addition, detailed measurements in low template concentrations proved a one-to-one correspondence between each Δ*I*(*t*) excursion and one dNTP incorporation. The dissociation constant *K*_D_ for the KF-template complex is 5 nM [94,95], and template concentrations below 1 nM increase the likelihood for KF to be unbound. Under these conditions, Δ*I*(*t*) showed quiet periods in which KF waited for template association interrupted by a cluster of continuous Δ*I*(*t*) excursions (Figure 4a). Still lower concentrations like 0.1 nM reduced KF’s average rate one hundred fold; in this limit, diffusional waiting times grew to many seconds but the instantaneous processing rate within a cluster of events remained 20 s^−1^. Thus, Δ*I*(*t*) was determined to accurately identify the arrival, processing, and dissociation of individual template strands.

In this low-concentration limit, the number of Δ*I*(*t*) excursions observed for each template molecule could be enumerated and compared to the template’s overhang length. Figure 4b illustrates a low-resolution histogram of such counts peaked around 42 Δ*I*(*t*) excursions for a homopolymer template (dC)_42_ [49]. Some template molecules dissociate before KF reaches the end of the strand, giving clusters with fewer than 42 counts; but very few clusters registered more than 42 counts, indicating that the event filtering in Figure 3 correctly identifies the base incorporation events. Δ*I*(*t*) can therefore be interpreted as a direct record of KF as it waits for template to arrive, processes the template base-by-base at its normal rate, and then dissociates from the template.

Having determined that individual Δ*I*(*t*) excursions correspond to single nucleotide incorporations, the individual low- and high-current levels in *I*(*t*) can be assigned to two portions of KF’s catalytic cycle. Since nucleotide incorporation occurs during a brief closing of the fingers subdomain [80], the Δ*I*(*t*) excursion is assigned to some portion of the fingers-closed conformation. The baseline *I*(*t*) level, on the other hand, is associated with KF’s rate-limiting open conformation. According to X-ray crystallography, the fully-closed conformation can only be accessed when template and complementary nucleotide are both bound to KF [85]; this selectivity helps explain why Δ*I*(*t*) excursions correctly enumerate template lengths without erroneous extra counts from random fluttering motions or the rejections of non-complementary nucleotides.

The X-ray crystal structures also reveal the precise motions of charged amino acid residues surrounding the 790C attachment site. The allosteric motions of these charges, which are essentially adjacent to the SWNT-FET, are primarily responsible for electrostatically gating the sensitized region of the SWNT and inducing the observed Δ*I*(*t*) signal. The motions of KF’s more distant charges and charged domains are screened by the surrounding buffer, which has a Debye screening length of 1 nm [96]. In similar single-molecule SWNT-FET measurements using T4 lysozyme instead of KF, charged residues near the attachment site were varied by mutagenesis to prove their role in signal transduction [47]. Therefore, the SWNT-FET is not merely a precise transducer of enzyme motion; the system can be designed to probe selected subdomain motions with appropriate design of the attachment site and nearby charged residues.

**Figure 5:**
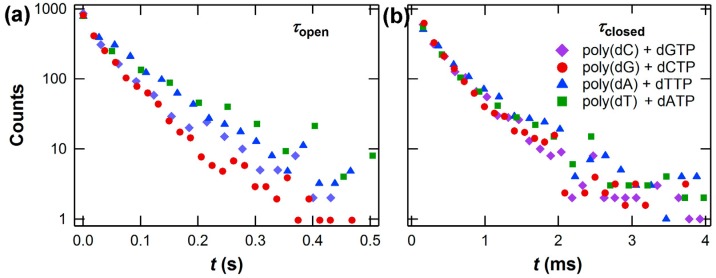
Example distributions of (**a**) *τ*_open_ and (**b**) *τ*_closed_ acquired for all four native nucleotides using a single KF molecule. The different slopes for *τ*_open_ represent different average processing rates for the four nucleotides. The portion of the cycle represented by *τ*_closed_, in which catalytic incorporation of the nucleotide occurs, is insensitive to the nucleotide species. The exact value of <*τ*_open_> varies from one molecule to another and can be 50% to 300% of the values shown here. However, each different copy of KF follows the trends shown here.

Identifying the levels in *I*(*t*) as open and closed conformations enabled a statistical analysis of KF’s single-nucleotide incorporation kinetics. Each dNTP incorporation was represented by one Δ*I* excursion, which had a duration *τ*_closed_ and a waiting time *τ*_open_ during which KF was in its open conformation. Figure 5 shows *τ*_closed_ and *τ*_open_ probability distributions from thousands of incorporation events for each of the four homopolymeric DNA templates measured with a significant excess (10 μM) of complementary dNTPs. The combination of eight *τ* distributions gives a sense of the information density of the SWNT-FET technique and the opportunities for revealing new information about molecular processes and molecule-to-molecule variation. The distributions are summarized in Table 1 for one typical KF molecule.

The kinetic results showed that the rate-limiting duration <*τ*_open_> was much longer than <*τ*_closed_>, averaging 50 ms. In addition, <*τ*_open_> was sensitive to the specific nucleotide, being nearly twice as long for dTTP and dATP incorporations than for dCTP and dGTP incorporations. The longer duration for A·T base pairs compared to C·G base pairs, regardless of which nucleotide is being incorporated into the single-stranded template, suggests that the formation of A·T pairs may involve a mechanism that is distinct from the formation of C·G pairs. Two different conformations, for example, could be relevant for dNTP recognition and processive fidelity. Unlike <*τ*_open_>, the mean duration <*τ*_closed_> was insensitive to dNTP identity with values of 0.3 to 0.4 ms. Thus, the events sensed by the SWNT-FET represent a brief, efficient part of the enzyme’s closure or closed configuration [87], occurring with a timing that is independent of the specific nucleotide. When averaged together to produce an effective replication rate *k* = (<*τ*_closed_> + <*τ*_open_>)^−1^, the SWNT-FET measurements are consistent with other published values for KF’s activity [77,78,91].

In addition to durations and kinetics, one more independent aspect of the Δ*I*(*t*) excursions is the signal amplitude. Since Δ*I*(*t*) is transduced by motions of the protein’s charge residues, differences in Δ*I*(*t*) are an indirect means of probing the conformational differences associated with the formation of different base pairs. On average, A·T base pairs produced larger Δ*I*(*t*) excursions than C·G base pairs. As with the timing, however, differences did not distinguish dATP incorporations from dTTP incorporations.

**Figure 6:**
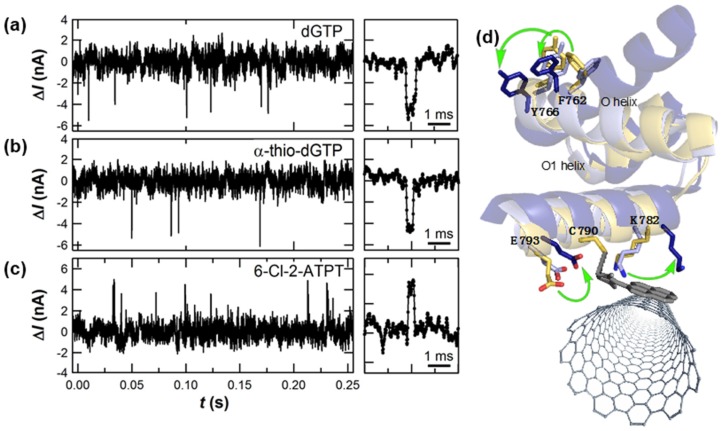
Direct comparison of nucleotide incorporation events into poly(dC)_42_ templates using: (**a**) native dGTPs; (**b**) α-thio-dGTP; and (**c**) 6-Cl-2-APTP. (**d**) Reversal of the excursion direction suggests that conformational accommodation of the latter analog is allosterically transmitted to the SWNT, perhaps through motions of the O-helix depicted here.

Even larger conformational effects were observed when a native dNTP was replaced with a synthetic, unnatural dNTP analog. Long-duration measurements of the same KF molecule allowed direct comparisons of dCTP incorporation against dCTP analogs such as α-thio-dCTP or 2-thio-dCTP, molecules for which slight alterations slowed down or sped up KF’s average kinetics, respectively (Figure 6) [50]. The Δ*I*(*t*) amplitude for 2-thio-dCTP also revealed a bistability in which KF adopts one of two different conformations during fidelity checking of the nucleotide incorporation. This effect was even more pronounced with 6-chloro-2-aminopurine-drTP (6-Cl-2-APTP), a pseudo-analog of dGTP that replaces the 6-amino group with chlorine to dramatically decrease the hydrogen bonding of the new base pair. During 6-Cl-2-APTP incorporations into homopolymeric poly(dC) templates, Δ*I*(*t*) excursions had *τ*_closed_ durations similar to the native dGTP but with reversed magnitudes, as shown in Figure 6c [50]. The reversal of Δ*I*(*t*) indicated different motions of charged residues as KF accommodated the unnatural analog. We have proposed that the active site O*-*helix may twist in two different directions for dGTP and 6-Cl-2-APTP. Rotations of Y766 and F762 in the active site, depicted in Figure 6d, can allosterically propagate to charged residues adjacent to the SWNT-FET and cause the observed signals. In fact, a brief conformational change has been observed by smFRET when KF moves the nascent base pair to a post-insertion site [77], and this motion may be the conformation sensed during *τ*_closed_. A twisting O*-*helix mechanism would also account for the larger Δ*I*(*t*) excursions for A·T base pairs, which bury deeper in KF’s active site [97] and allow greater motions of the O-helix.

### 3.2. Accuracy of Amplification-Free Measurements of DNA Template Lengths

The previous section described how SWNT-FET devices have provided detailed recordings of DNA polymerase activity. Despite being densely informative, the Δ*I*(*t*) signals are currently insufficient for DNA sequencing applications because they do not fully differentiate between the four base pairs. Successful differentiation will require additional work tailoring the transduction to be more sensitive to small differences between the bases. Nucleotide analogs already show the promise of this strategy in Figure 6, and targeted point mutations may further enhance the SWNT-FET’s sensitivity to specific nucleotide-recognition mechanisms of KF.

To justify further work in those directions, experiments revisited the conclusion that Δ*I*(*t*) successfully records every base incorporation. Accurate base enumeration, after all, is a critical precondition to successful sequencing, and the first counting experiments using (dC)_42_ were not designed to determine the method’s accuracy. To investigate base enumeration in greater detail, we repeated the diffusion-limited length counting experiments described for Figure 4 using DNA templates designed to assess the challenges of a (CTTT)_n_ repeating motif. (CTTT)_n_ occurs with 80 different alleles on chromosome 4 of the human alpha fibrinogen gene, and this variety is potentially useful for human identification from DNA [98]. In fact, traditional DNA sequencing-by-synthesis technologies have difficulty determining the correct length of this and other repeated motifs in the human genome, and accurate base-by-base readout with SWNT-FETs represents a new opportunity to correctly enumerate these segments. In addition, in vivo errors by polymerase as it replicates repeating motifs lead to disease and cellular malfunction; so, accurate base-by-base readout might reveal the causes of such errors.

Improved counting experiments used four templates designed with 9, 10, 11, and 26 repeats of the sequence CTTT. For comparison, experiments also used random sequences with the same length like (B_10_A)_4_, in which B_10_ represents a random sequence of G, T, and C. As described above, KF-functionalized SWNT-FETs were measured for 10 min in template solutions diluted to 0.5 nM to produce diffusion-limited waiting times of multiple seconds, on average, between each template strand. At the end of each idle waiting time, template association initiated a cluster of stochastic Δ*I*(*t*) excursions that occurred at an average rate of 20 s^−1^. The events in each cluster were enumerated and binned into histograms, and the template concentration was kept low enough that no double-length clusters from two, back-to-back template molecules were observed.

To minimize bias in the data processing, a semi-automated routine was used to identify and count Δ*I*(*t*) excursions. After smoothing the raw data to remove baseline fluctuations, the routine determined the standard deviation σ of the *I*(*t*) baseline noise and then counted events that met two criteria: magnitudes greater than 3σ and durations longer than 100 µs (10 data points at 100 kHz sampling rates). Figure 3 illustrates an Δ*I*(*t*) record with example events accepted and rejected by these dual criteria. Small-magnitude and short-duration fluctuations that were rejected by the filter may have represented real motions of KF intermediates, but studying such transients accurately and in greater detail will require future measurements with higher bandwidths. This work focused exclusively on events accepted by the filter and, specifically, identifying the long pauses used to define clusters of events. The pause between two adjacent events within a cluster was only 80 ms on average, so idle baseline lasting >0.5 s was deemed an extraordinary duration indicative of template dissociation.

**Figure 7:**
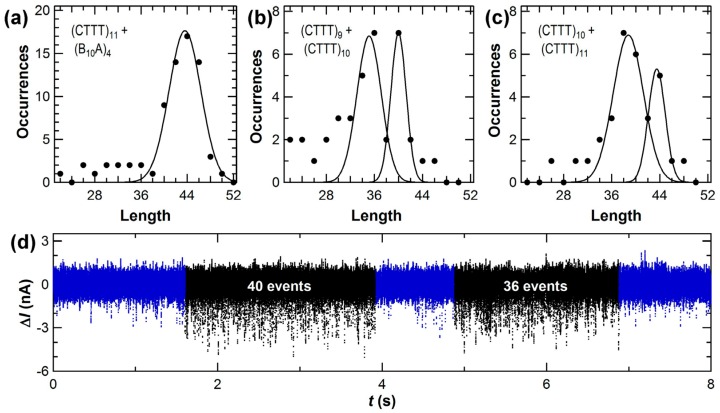
Base-enumeration distributions obtained from 0.5 nM concentrations of the templates: (**a**) (CTTT)_11_ and (B_10_A)_4_ measured separately; (**b**) (CTTT)_9_ mixed with (CTTT)_10_; and (**c**) (CTTT)_10_ mixed with (CTTT)_11_. Solid curves are Gaussian curve fits to each peak and fitting results are summarized in Table 2. Mixing two templates together led to fewer that 25 template molecules being counted in a typical data record, but the accuracy of peak fitting was not substantially reduced. (**d**) Example Δ*I*(*t*) record collected with the (CTTT)_9_ + (CTTT)_10_ mixture, illustrating a cluster of 40 dNTP incorporations, a 1-s pause, and a cluster of 36 incorporations.

Figure 7 shows new length-enumeration histograms obtained with the SWNT-FET technique. A template like (CTTT)_11_ produced 44 stochastic events over a duration of 2 to 4 s, followed by one or more seconds of idle baseline noise (Figure 7d). Over the course of 10 min acquisition periods, fewer than 40 templates were typically bound, processed, and released by KF. Despite the low number of molecules, the distribution could be fit by a single Gaussian peak. Furthermore, measurements with template mixtures produced enumeration histograms having separately resolved peaks. Results from a 1:1 molar ratio of (CTTT)_9_ with (CTTT)_10_ are shown in Figure 7b, and the mixture of (CTTT)_10_ with (CTTT)_11_ is shown in Figure 7c. In both pairs, the templates differ by just one repeat unit of four base pairs (bp). The total template concentration was kept constant in each experiment, so the distributions with template mixtures contained fewer than 20 molecules of each template in most data records. The uncertainty in peak position was ≤0.3 bp in each fit, and other fitting results are summarized in Table 2. With template mixtures, the peak position errors were no worse than in the single-template case, indicating that template molecules did not interfere with each other at these concentrations. In Figure 7d, a data record shows a (CTTT)_10_ template and then a (CTTT)_9_ being processed in succession to illustrate how KF’s processing of one molecule in a mixture was independent of the next.

Two main sources of error were identified during the analysis of these measurements. First, the peak positions determined by Gaussian fitting were systematically smaller than the actual template length, with an average of −0.6 bp. This error resulted from the 2-bp width of the bins needed for analyzing so few counts. Second, the peaks exhibited a full-width at half maximum (FWHM) of 6 to 8 bp. The FWHM primarily arose from isolated events occurring during the 0.5 s before and after each cluster. These events, which could not be conclusively associated with a template, led to uncertainties as large as ±2 bp in the enumeration of the worst-case data clusters. In other words, the overall accuracy in peak position reached <1 bp after as few as 20 template reads, but the length of any single template molecule included errors of ±2 bp.

Accuracy can be improved somewhat with larger data sets. Our experimental protocol flushed or rinsed the devices with buffer every 10 min to avoid excess contamination from non-specific adsorption. However, consecutive data sets from a single device were no better nor worse than nonconsecutive data from different days or data appended from two or more different SWNT-FET devices. Consequently, several data sets could be accumulated to compile larger numbers of template reads for improved analysis of template mixtures. For example, the repeating motif (CTTT)_11_ was read just as accurately as the semi-random sequence (B_10_A)_4_, so these two distributions have been combined in Figure 7a. The single peak indicates that the SWNT-FET technique was insensitive to the repeating sequence, though the peak width is slightly larger than peaks shown in Figure 7b,c. In another experiment, a similar distribution was accumulated from 200 min of single-molecule signal containing nearly 1000 template reads. With a bin size reduced to 1 bp, the systematic error in peak position was reduced from −0.6 bp to −0.2 bp and the uncertainty in the Gaussian fit reduced from 0.3 bp to 0.2 bp. Even with these larger data sets, the FWHM only reduced to 4 bp because the errors associated with correct identification of the beginning and end of each template molecule do not average out. Better accuracy in single-molecule enumeration will require better identification of these endpoints, either by lowering the template concentration further or by developing techniques that create other distinguishing features in *I*(*t*).

The measurements focused on templates shorter than 50 base pairs, but there was no evidence suggesting that longer templates would fare worse. In fact, the FWHM was not proportional to template length since it primarily arose from the template endpoints. Thus, long templates could be read with the same accuracy as short templates, and improving the analysis methodology might further reduce the FWHM without requiring longer data records. On the other hand, we note a tendency of shorter templates to associate with KF more readily than longer templates. In the 1:1 mixtures depicted in Figure 7b,c, the different areas under each peak suggest that shorter templates were counted nearly twice as often as longer templates. The exact magnitude of this difference also depended on the sequences used. The reduction may reflects different diffusion and KF-association constants for the two template molecules; it also illustrates that this technique can be inaccurate for determining molecular ratios.

In general, the templates described here were chosen to be shorter than KF’s maximum processivity. Previous ensemble measurements have estimated this processivity limit to be 50 ± 5 bp [89,90], but the value is very difficult to determine precisely. The SWNT-FET technique provides a new, direct method for measuring processivity. Using the (CTTT)_26_ template with an overhang length of 104 bp, we have observed rare sequences of up to 94 Δ*I*(*t*) excursions but none as long as the template. Instead, the distribution of cluster lengths had no peak and averaged only 70 bases, indicating that KF dissociates from the template before reaching its end. The distributions in Figure 7 provide a good measurement of this dissociation probability because the non-zero background on the left of each histogram peak represents those cases where KF dissociated mid-template before reaching its end. Within the limits of the available statistics, this background was constant from the template length down to ~5 bp (below which it becomes difficult to distinguish short templates from random noise and spurious events), indicating a constant dissociation probability after each base incorporation. Based on the fraction of background events in each bin of the histogram, we calculate this probability to be 1.8% ± 0.2%. A probability of ~2% agrees with the ensemble processivity of about 50 bp [89,90], while also allowing for the rarer 70 and 80 bp runs observed with (CTTT)_26_. It is also possible that enhanced processivity results from an unintended stabilization of the template-KF complex by the SWNT. In all other respects, the SWNT-FET measurements agree with ensemble measurements of KF activity.

Note that the background counts used for estimating the dissociation rate were not observed to the right of each peak, since the templates had a finite length. In fact, the absence of excess counts illustrates the efficiency of dNTP incorporations with each closure. If more than one closure was needed for KF to successfully position the template strand and incorporate a nucleotide, then the histograms shown in Figure 7 would extend well beyond the template’s actual length. Other than the peak’s FWHM, extra counts beyond the template length have not been observed. Thus, the inefficient steps of recognizing the correct dNTP and rejecting mismatched ones do not register as events in this SWNT-FET signal, and such mechanisms must be occurring during *τ*_open_. The same conclusion has been reached by smFRET studies and it has been used to explain why *τ*_open_ is the rate-limiting portion of the catalytic cycle [80,83].

## 4. Conclusions

Single-molecule enzymology provides many new opportunities to understand complex biochemical mechanisms. While single-molecule optics have become a well-established characterization method, single-molecule electronics have only recently been demonstrated. Here, we have summarized one successful method for building and using single-molecule electronic devices. Using KF as a model enzyme with a complex catalytic cycle, we have demonstrated high resolution, long-duration electronic recording that reveal KF’s sensitivity to native and analog nucleotides and its processivity limits. Selection of an appropriate attachment site maximized the device’s sensitivity to nucleotide incorporations over other protein movements, enabling error-free base-pair enumeration unaffected by nucleotide mismatch rejections. While these particular results with KF have been described in relation to DNA sequencing applications, they represent a much more general method for single-molecule enzymology that is readily extended to other processive enzymes.

In that sense, single-molecule electronics is likely to be complementary to traditional single-molecule fluorescence. The electronic technique was well-suited to the question of processivity, for example, and it was straightforward to continuously monitor one molecule’s activity in many different conditions. More general benefits of the solid state platform include new opportunities for parallelization in arrays and high bandwidth detection of brief fluctuations. In addition, new research becomes possible for proteins that do not readily incorporate fluorophores or which are especially sensitive to photo-oxidation. The availability of single-molecule electronics is unlikely to replace other single-molecule techniques, but it does provide a new tool for addressing difficult research questions. In fact, since the electronic readout is independent of optical excitation, future devices may even provide opportunities to combine the electronic and fluorescent channels for dual, simultaneous readout of two subdomains of the same molecule. That type of synergetic combination prompts hope that creative researchers will find many uses for single-molecule electronics as a research platform.

## Figures and Tables

**Table 1:** Single-nucleotide processing rates with homopolymeric templates ^1^.

Template	Nucleotide	*τ*_closed_ (ms)	*τ*_open_ (ms)	*k* (1/s)
poly(dT)_42_	dATP	0.33 ± 0.08	71.4 ± 1.4	14.4 ± 2.9
poly(dA)_42_	dTTP	0.42 ± 0.09	63.7 ± 1.1	16.0 ± 2.9
poly(dG)_42_	dCTP	0.32 ± 0.07	39.0 ± 5.6	26.2 ± 4.4
poly(dC)_42_	dGTP	0.33 ± 0.05	38.0 ± 5.8	28.5 ± 3.5

^1^ Average values ± one standard deviation.

**Table 2:** Gaussian fitting parameters from length-counting experiments.

Template Sequence	Length(s)	*N* ^2^	Peak Position(s) (bp)	FWHM (bp)
(CTTT)_11_	44	27	42.3 ± 0.3	7.6 ± 0.8
(B_10_A)_4_	44	45	44.2 ± 0.1	6.4 ± 0.4
(CTTT)_11_ and (B_10_A)_4_	44 and 44	72	43.6 ± 0.2	7.8 ± 0.6
(CTTT)_9_ and (CTTT)_10_	36 and 40	38	34.5 ± 1.1 40.0 ± 0.2	10.1 ± 3.6 3.6 ± 0.4
(CTTT)_10_ and (CTTT)_11_	40 and 44	31	38.8 ± 0.3 43.5 ± 0.3	7.1 ± 1.0 4.0 ± 1.0

^2^ Total number of template molecules observed in the distribution.

## Abbreviations

The following abbreviations are used in this manuscript:

KF	Klenow Fragment of DNA polymerase I
DNA	deoxyribonucleic acid
smFRET	single-molecule Förster resonance energy transfer
SWNT	single-walled carbon nanotube
FET	field effect transistor
dNTP	deoxynucleoside triphosphate
